# Female reproductive ageing persists despite high infanticide risk in chacma baboons and geladas

**DOI:** 10.1098/rsos.241210

**Published:** 2025-01-15

**Authors:** Jacob A. Feder, India A. Schneider-Crease, Jacinta C. Beehner, Thore J. Bergman, Robert M. Seyfarth, Joan B. Silk, Noah Snyder-Mackler, Amy Lu

**Affiliations:** ^1^ Institute of Human Origins, Arizona State University, Tempe, AZ, USA; ^2^ School of Human Evolution and Social Change, Arizona State University, Tempe, AZ, USA; ^3^ Center for Evolution and Medicine, Arizona State University, Tempe, AZ, USA; ^4^ Department of Psychology, University of Michigan, Ann Arbor, MI, USA; ^5^ Department of Anthropology, University of Michigan, Ann Arbor, MI, USA; ^6^ Department of Ecology and Evolutionary Biology, University of Michigan, Ann Arbor, MI, USA; ^7^ Department of Psychology, University of Pennsylvania, Philadelphia, PA, USA; ^8^ School of Life Sciences, Arizona State University, Tempe, AZ, USA; ^9^ Department of Anthropology, Stony Brook University, Stony Brook, NY, USA

**Keywords:** reproductive senescence, maternal effects, sexually selected infanticide, folivory

## Abstract

Across mammals, fertility and offspring survival are often lowest at the beginning and end of females’ reproductive careers. However, extrinsic drivers of reproductive success—including infanticide by males—could stochastically obscure these expected age-related trends. Here, we modelled reproductive ageing trajectories in two cercopithecine primates that experience high rates of male infanticide: the chacma baboon (*Papio ursinus*) and the gelada (*Theropithecus gelada*). We found that middle-aged mothers generally achieved the shortest interbirth intervals in chacma baboons. By contrast, old gelada females often showed shorter interbirth intervals than their younger group-mates with one exception: the oldest females typically failed to produce additional offspring before their deaths. Infant survival peaked in middle-aged mothers in chacma baboons but in young mothers in geladas. While infant mortality linked with maternal death increased as mothers aged in both species, infanticide risk did not predictably shift with maternal age. Thus, infanticide patterns cannot explain the surprising young mother advantage observed in geladas. Instead, we argue that this could be a product of their graminivorous diets, which might remove some energetic constraints on early reproduction. In sum, our data suggest that reproductive ageing is widespread but may be differentially shaped by ecological pressures.

## 1. Introduction

Reproductive output, which includes both fertility and offspring viability, typically changes across the lifespans of female animals [1,2]. Young individuals generally exhibit poor reproductive outcomes, perhaps because they must balance finishing growth with commencing reproduction (e.g. [3,4], reviewed in [5]), while old individuals undergo reproductive senescence as they experience somatic decline [6–8]. These shortfalls at both extremes of the reproductive lifespan have been theoretically supported by models of life history evolution [9,10], empirically demonstrated in model systems (e.g. *Caenorhabditis elegans*: [11], *Drosophila melanogaster*: [12]), and suggest that reproductive ageing generally follows a ∩-shaped pattern (offspring survival: [1], fecundity: [2]).

However, not all species and populations conform to this pattern. When females delay breeding until growth is nearly complete, reproductive performance often peaks shortly after maturation and declines as mothers age (e.g. arthropods: [13,14]). By contrast, in taxa where ecological knowledge (e.g. elephants: [15], grey seals: [16]), maternal experience (e.g. horses: [17]) or maternal energetic resources (e.g. turtles: [18], pinnipeds: [19]) improve with age, older mothers can maintain high reproductive success. Reproductive ageing can also be obscured if the recruitment of non-breeding helpers [20,21] or compensatory increases in maternal care (i.e. ‘terminal investment’: [22]) offset age-related declines in maternal condition, or exacerbated if ecological challenges disproportionately impact the youngest or oldest individuals (e.g. seabirds: [7,23]). Given this wide variation in patterns of reproductive ageing, the ecological and evolutionary factors shaping reproductive ageing remain poorly understood.

Life history datasets from long-term, individual-based studies have increasingly enabled researchers to capture age-related changes in reproductive performance (reviewed in [24]). Many of these reproductive ageing studies have focused on non-human primates (e.g. [25,26]), given their ‘slow’ life histories [27] and a long history of individual-based research [28]. In a recent analysis of reproductive ageing patterns across seven non-human primate species, all but one exhibited ∩-shaped or negative ageing trajectories [29]. Capuchins showed no changes in reproductive rates or infant survival as females aged, which was attributed in part to extreme droughts [30] and alpha male takeovers [31]—stochastic, unpredictable events linked with increases in infant mortality risk (including male infanticide). In other words, these salient extrinsic challenges could obscure reproductive ageing patterns by adding age-independent variance to reproductive outcomes. Because variably harsh environments and high rates of male infanticide strongly impact female reproductive success across many primate taxa (e.g. *Alouatta seniculus*: [32,33], *Propithecus edwardsi*: [34,35]), similarly negligible ageing effects could be more widespread than previously reported. Nevertheless, reproductive ageing can persist in populations facing extreme weather events (e.g. *Uria aalge*: [7]) and high infanticide rates (e.g. *Panthera leo*: [36]). If so, then variation in ecological pressures may better explain the presence and intensity of ageing effects across species.

Here, we tested the relationship between age and reproductive performance in the chacma baboon (*Papio ursinus*) and the gelada (*Theropithecus gelada*), two cercopithecine primates that share infanticide as a primary source of offspring mortality and have been the subject of long-term study [37,38]. Chacma baboons form large, cohesive social groups [39], while geladas live in multi-level societies in which several small, one-male ‘units’ range together to form herd-like ‘bands’ [40]. Dominant male tenures are also shorter in the multi-male groups of chacma baboons [41] than in the one-male units of geladas [42]. Given less frequent and more predictable patterns of alpha male turnover, female geladas experience lower overall rates of offspring mortality [37,38] and implement many counterstrategies that can further pre-empt infanticide (e.g. fetal loss, or the ‘Bruce Effect’: [43]). Additionally, predation—another presumably age-independent challenge—is a critical source of mortality in chacma baboons [38] but not geladas [40]. Thus, while infanticide risk might obscure quantitative signals of reproductive ageing in both species, the weaker extrinsic mortality risks observed in geladas might allow ageing effects to weakly persist (the infanticide hypothesis, table 1).

**Table 1.** Expected maternal ageing effects in our study species across our two main hypotheses.

species	infanticide hypothesis	ecological hypothesis
chacma baboons	no observable effects	strong effects
geladas	weak-to-absent effects	weak effects

Alternatively, ageing patterns might more closely reflect ecological and dietary pressures. In general, folivorous primates produce proportionally smaller infants [44,45] and experience weaker forms of feeding competition [46,47]. Given the variable costs diet might thus impose on reproduction, ageing patterns could instead be strong in chacma baboons, which are omnivorous and face year-round contest competition over limited food resources [39,48], but weak in geladas, which mostly eat grasses and engage in contests primarily during a short dry season [49]. However, while diet has been both theoretically and empirically linked with cross-species variation in life history traits [50–52], its influence on the pace of ageing remains unclear (but see [53]). Because chacma baboons face harsher environmental conditions and show greater variation in offspring survival than geladas [37,38], but see [54], we may expect them to show stronger age-related changes in reproductive performance (the ecological hypothesis, table 1).

To quantify ageing patterns in these species and evaluate these hypotheses, we combined demographic records from two long-term study populations of chacma baboons and geladas and modelled female reproductive ageing using complementary strategies. First, we constructed life tables to document differences in age-specific fertility and survivorship across the two species. Using these life tables, we also evaluated whether these species experience quantitatively discernible post-reproductive periods. Second, we implemented survival analyses to determine whether maternal age predicted (i) the duration and closure of interbirth intervals and (ii) the probability of infant survival. In combination, these datasets and approaches allowed us to evaluate whether reproductive ageing is (i) obscured by infanticide in part or in full (the infanticide hypothesis) or (ii) persistent across species but more salient in populations that are prone to nutritional shortfalls (the ecological hypothesis).

## 2. Methods

### 2.1. Study sites and populations

Data on chacma baboons were collected from a wild population at the Moremi Game Reserve, Botswana. Between 1992 and 2007, researchers collected behavioural and demographic data from a social group that in any given month included 4–15 adult males, 18–28 adult females, and their dependent offspring. For this study, we used demographic data from 58 females who gave birth at least once during the study period. Data on geladas were collected from a wild population in the Simien Mountains National Park, Ethiopia. Between 2006 and 2022, the Simien Mountains Gelada Research Project collected routine behavioural and demographic data from 46 social units (20 founding units and 26 units that formed via fission and fusion events), each containing one leader male, 0–4 follower males, 1–12 adult females, and their dependent offspring. For this study, we used demographic data from 176 females who gave birth at least once.

### 2.2. Demographic data

For chacma baboons, research from a previous study period (1977−1991) allowed dates of birth to be known within one month for all but three mothers whose ages were estimated as juveniles. For geladas, dates of birth prior to the study period were unknown. Thus, maternal ages were determined from a combination of known birth dates (*n* = 49) and estimates drawn from life history information (*n* = 127). Analyses incorporating uncertainty in these age estimates were congruent with those using fixed estimates for maternal age (see electronic supplementary material, supporting information). Thus, we report analyses using these fixed estimates here.

We assessed reproductive performance across the lifespan of mothers using data on infant births (n_chacmas_ = 191; n_geladas_ = 453) and deaths (n_chacmas_ = 71; n_geladas_ = 79). Given near-daily observations at the study sites, births were unlikely to have been missed. For chacma baboons, all infant births were known within a two-week window. For geladas, most infant births were also known within this interval (*n* = 408). When gelada infant births were not known with this accuracy (*n* = 45), dates of birth were assigned using the infant’s face coloration (which fades from pink to grey within the first month of life) and coat coloration (which changes from black to brown by six months of age: [55]). Interbirth intervals were then calculated as the time difference between successive births. Here, we only used intervals following surviving offspring, as infant deaths hasten subsequent births [56,57]. For chacma baboons, our sample included 83 closed intervals and 20 that were right-censored when mothers died or when observations ended. For geladas, our sample included 240 closed intervals and 93 right-censored intervals.

Since we removed cases where interbirth intervals were extrinsically shortened by male infanticide, the remaining dataset should be well-poised to capture intrinsic, age-related changes in maternal condition and the resulting pace of reproduction. However, this reduction in sample size might also reduce statistical power to capture ageing effects [29]. Moreover, even after removing birth intervals associated with infant deaths, male geladas may still play a role in shaping these intervals. As noted above, pregnant geladas experience fetal loss when new males enter their social units [43]. Thus, we conducted additional analyses that accounted for the effects of male takeovers on these interbirth intervals in geladas (electronic supplementary material, figure S1 and table S2, supporting information), which are concordant with the results provided in the main text.

For both study populations, we assessed infant survival up to 1 year of age. This allowed us to match the analyses provided in Campos *et al.* [29] and focus on the developmental window when infanticide risk is highest [38,42]. Although infant deaths were rarely observed directly, we were able to attribute many infant deaths to (i) maternal death, (ii) infanticide and (iii) injury or illness based on the following criteria. For both species, infant deaths were classified as resulting from maternal death if they occurred within the 90 days following their mother’s disappearance. For chacma baboons, deaths were classified as infanticides when infants died following an observed male attack or when such attacks were indirectly inferred from injuries to the offspring or mother. For geladas, deaths were classified as infanticides if they occurred within 180 days following a male takeover (*n* = 101 takeovers), following [37]. Deaths were classified as resulting from injury or illness if infants were seen with an injury or exhibiting clear signs of ill health in the month leading up to their deaths. Predation events were never reported for infants in either population, although some infant deaths that co-occurred with maternal disappearance may have directly or indirectly resulted from predation. All remaining infant deaths were attributed to (iv) unknown causes. While unknown deaths were associated with circumstantial evidence (e.g. harsh weather events), corroborating information was too scant to create more fine-grained categories.

### 2.3. Life table construction and analysis

To describe and contrast life history patterns in female chacma baboons and geladas, we constructed life tables using the demographic data described above. Life tables divided all births, deaths and censoring events into yearly intervals (0−1 yr, 1−2 yr, etc.). Females entered the life table upon their birth or the onset of their group’s study and exited the life table upon their death or at the end of the study period. For this analysis, we focused on two metrics: *l_x_*, which notes the probability that a female survived from birth to age *x*; and *m_x_*, which notes the probability that a female gave birth during the interval at age *x*. Unlike the survival analyses below, this approach did not exclude cases where fertility was hastened by offspring death. To identify ageing trends within our study species, we constructed two Bayesian generalized models with binomial outcomes using the ‘R’ package ‘brms’ [58]. In these models, we counted the number of (model 1) births and (model 2) deaths within each age class as the outcome while accounting for the total number of individuals included in that age class. Female age (linear and quadratic), population and their interaction were included as fixed effects.

Additionally, constructing life tables allowed us to calculate post-reproductive representation (PrR)—a metric that reflects the proportion of adult females that have ceased reproduction [59]. PrR is derived from the equation *T_M_* /*T_B_*, where *T_M_* indicates the expected number of years that a female will be post-reproductive and *T_B_* indicates the expected number of years that a female will be an adult. In this approach, females become ‘adults’ at the age class when 5% of lifetime fertility has been completed and become ‘post-reproductive’ at the age class when 95% of lifetime fertility has been completed. To assess whether PrR is non-zero, we compared our observed PrR measurements with those derived from 10 000 simulations in which the probability of reproducing and the probability of surviving decline in parallel during the years following peak fertility [59]. *P-*values were then calculated as the proportion of simulated PrR values that were greater than the observed PrR.

### 2.4. Statistical analyses

To assess the influence of maternal age on interbirth intervals, we constructed two hierarchical Bayesian survival models using ‘rstanarm’ [60], which modelled reproductive ageing effects using flexible M-splines and included maternal age and maternal age² as fixed effects. This approach allowed for the inclusion of right-censored cases, where mothers died or observations ceased before they gave birth to a subsequent offspring. Mothers entered the interbirth interval dataset once they reached the minimum observed interbirth interval duration in the population (chacma baboons: 0.99 yr; geladas: 1.10 yr). Because the effect of maternal age on interbirth intervals violated the assumption of proportional hazards, we included time-varying effects for maternal age and maternal age². We used default boundary and internal knots, allowing for smooth changes in the impact of maternal age as interbirth intervals progressed. Maternal identity, study year and (for geladas) social unit identity were included as random effects in all models.

To assess the relationship between maternal age and infant survival, we similarly constructed two Bayesian survival models including maternal age and maternal age² as fixed effects. Here, the proportional hazards assumption was not violated, so no time transformations were necessary. For both interbirth interval and infant survival models, we included maternal identity and study year as random effects, used default priors and fit models using four chains, each including 2000 iterations. For both the interbirth interval and infant survival analyses, we fit additional models that included maternal parity (i.e. primiparous vs. multiparous) as an additional predictor. However, these did not substantially improve model fit. Thus, we report results from the models using maternal age alone.

Furthermore, to assess whether these changes in infant survival across maternal age were driven by specific sources of infant mortality, we constructed additional Bayesian generalized linear mixed models for both species. Specifically, we constructed logistic models assessing whether maternal age and maternal age² predicted whether infants died due to (i) maternal death, (ii) infanticide, (iii) illness or injury or (iv) unknown causes, each coded as a 1/0 response. For these models, we restricted our datasets to include only infants whose fates were known, removing all infants who were right-censored before reaching 1 year of age. We included birth year as a random effect and used weakly informative priors (i.e. normal(0,1)).

## 3. Results

### 3.1. Life table patterns

**Figure 1.**
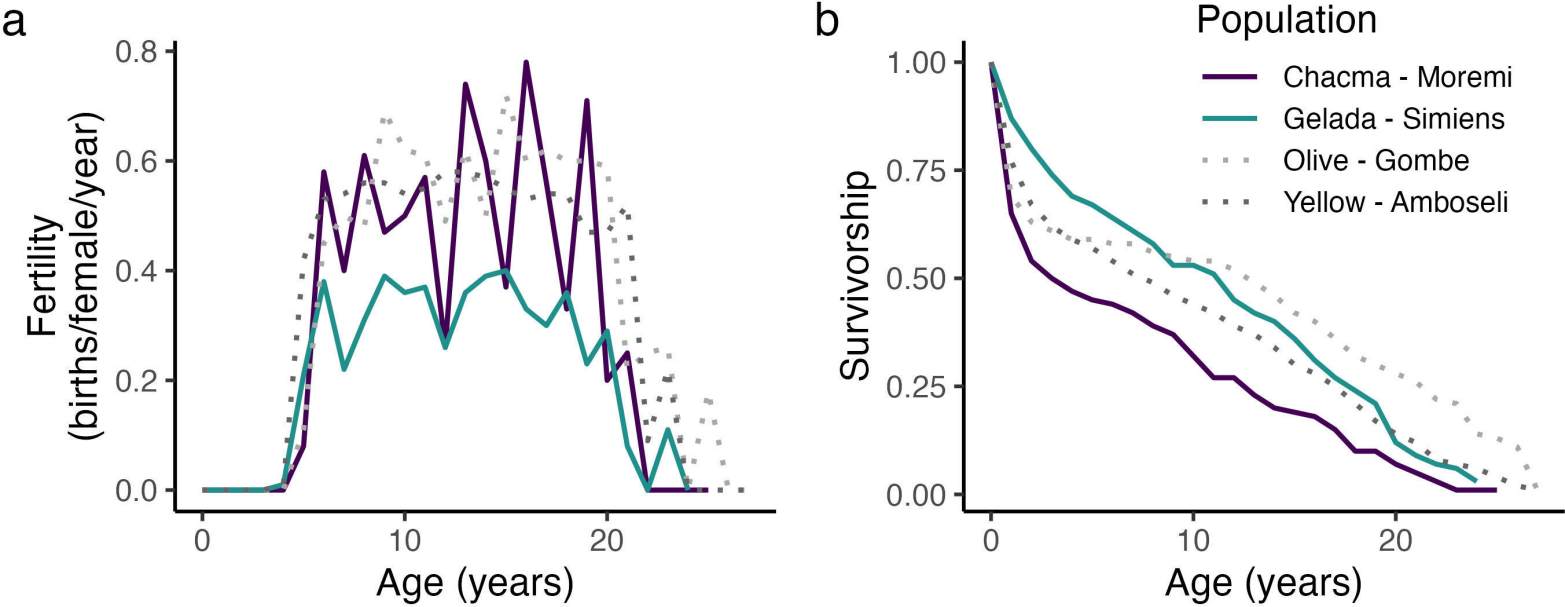
(*a*) Fertility and (*b*) survivorship across the lifespans of female chacma baboons and geladas. (*a*) Fertility (the proportion of females in each yearly age class that gave birth) rises upon entering adulthood and declines in old age in both chacma baboons (purple) and geladas (teal). (*b*) Survivorship is lower in chacma baboons, in large part due to higher rates of infant mortality. Published life table data from olive baboons (light grey, from [36] and [61]) and yellow baboons (dark grey, from [62]), which experience much lower rates of infanticide [63], are provided for additional comparative context.

For both chacma baboons and geladas, fertility increased at 4 years of age, mostly plateaued from approximately 6 to 20 years, and then declined in later life (posterior mean ± standard error: β_age_ = 2.41 ± 0.64; β_age2_ = −7.04 ± 0.61; figure 1*a*). Birth rates were generally higher in chacma baboons than in geladas (β_gelada_ = −0.76 ± 0.13), although chacma baboons began reproducing at later ages than geladas (chacma baboons: 6.89 ± 0.20 yr; geladas: 6.39 ± 0.09 yr). The fertility patterns identified for chacma baboons closely matched those identified in other long-term savannah baboon populations, while geladas showed much lower birth rates across their reproductive course (figure 1*a*). A chacma baboon female that survived to reproductive age gave birth to approximately 4.38 offspring, while a gelada female that did the same produced only 3.23 offspring over her reproductive lifespan.

In both chacma baboons and geladas, mortality rates peaked during infancy and again during later adulthood (β_age_ = 2.35 ± 0.65; β_age2_ = 3.30 ± 0.57). However, mortality rates were generally higher in chacma baboons than in geladas (β_species_ = −0.30 ± 0.15). This difference was largely driven by 1st-year mortality (35% vs. 13%), which is much lower in geladas than in both the chacma baboons and other savannah baboon populations (figure 1*b*). Given these differences, only 45% of chacma baboon females reached 5 years of age, compared with 71% of geladas. Thus, despite their faster reproductive rates, at birth, a chacma baboon female is expected to produce 1.98 offspring over her lifetime, while the average gelada female is expected to produce 2.23 offspring. For both chacma baboons and geladas, mortality rates rose again during old age (β_geladas x age_ = 2.30 ± 0.71; β_geladas x age2_ = 0.35 ± 0.64).

PrR was low in both chacma baboons (PrR_chacma_ = 0.044) and geladas (PrR_gelada_ = 0.051), and simulation tests indicate that these patterns did not deviate from null expectations (*P*_chacma_ = 0.15; *P*_gelada_ = 0.45). Thus, although fertility appreciably declines in old age, these changes parallel, and do not precede, increases in mortality risk.

### 3.2. Interbirth intervals

**Figure 2.**
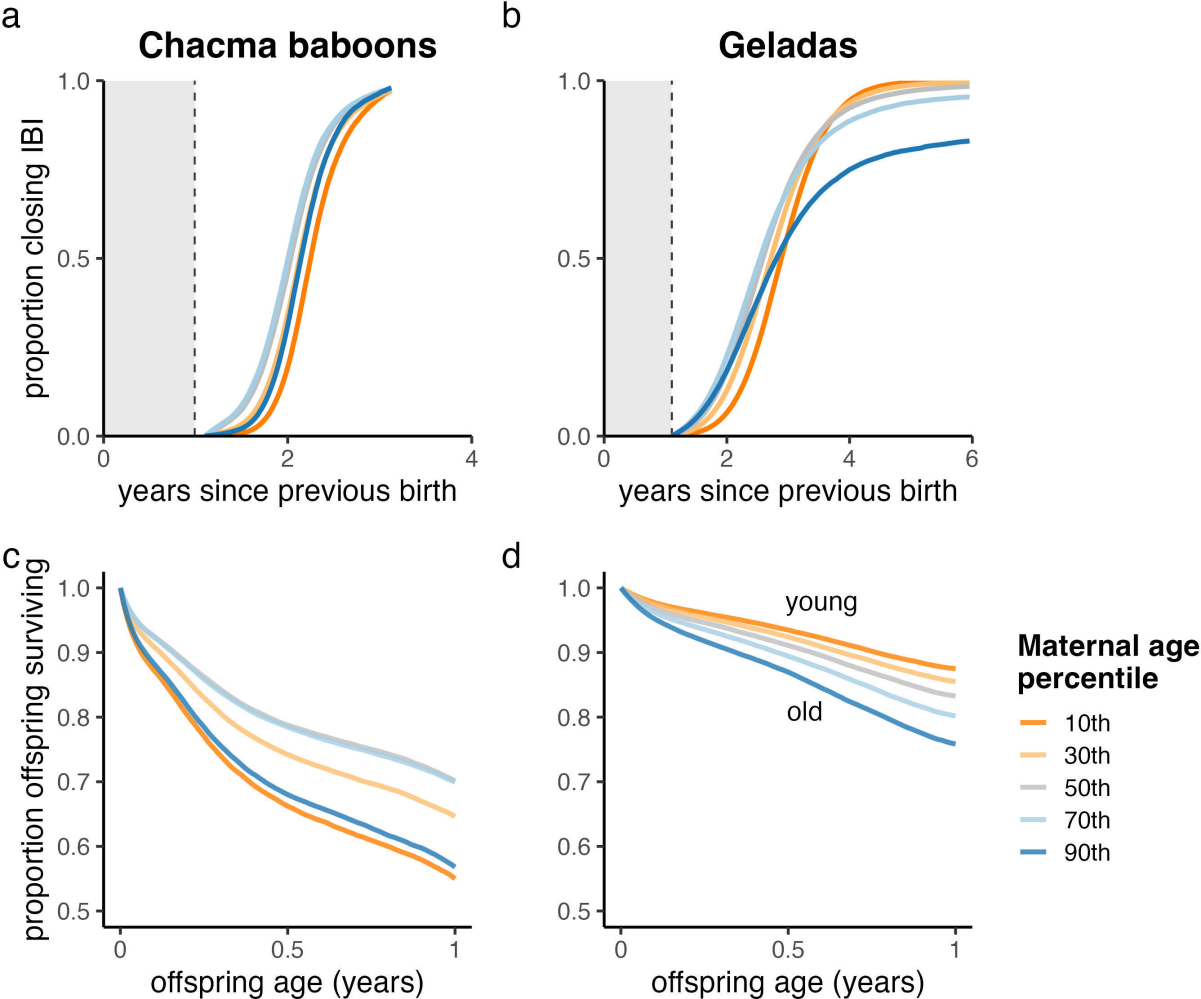
Effects of maternal age on reproductive outcomes in (*a,c*) chacma baboons and (*b,d*) geladas. (*a,b*) Interbirth intervals were generally longer in young and old chacma baboons and in young geladas. However, these trends disappeared or reversed once 2−3 years had passed, respectively. Old female geladas often failed to close very long interbirth intervals. (*c,d*) Infant survival was higher in middle-aged chacma baboons and in young geladas. Orange lines indicate young mothers, grey lines indicate mid-aged mothers, and blue lines indicate old mothers.

Closed interbirth intervals averaged 2.00 ± 0.36 yr in chacma baboons and 2.57 ± 0.66 yr in geladas. For both species, interbirth intervals were generally longer in younger mothers than in older mothers (chacma baboons: β_age_ = 0.80 ± 0.57 yr; geladas: β_age_ = 0.89 ± 0.31 yr; figure 2*a*,*b*). The effect of maternal age on interbirth intervals was quadratic in both chacma baboons and geladas (chacma baboons: β_age2_ = −1.72 ± 0.60; geladas: β_age2_ = −0.48 ± 0.23). However, while closed interbirth intervals were generally longer for both young and old chacma baboons, closed interbirth intervals were initially longer in young compared with old geladas. For chacma baboons, this quadratic relationship diminished as interbirth intervals grew longer, and credibility intervals for this effect eventually overlapped with zero (electronic supplementary material, figure S2a). For geladas, however, the effect of maternal age on the probability of closing an interbirth interval gradually reversed, as old mothers often failed to close long interbirth intervals (electronic supplementary material, figure S2b). In other words, although some old gelada mothers achieved short interbirth intervals, those that did not reproduce quickly often died before giving birth to another offspring. By contrast, almost all young mothers eventually gave birth again, even when interbirth intervals became very long.

### 3.3. Infant survival

**Figure 3.**
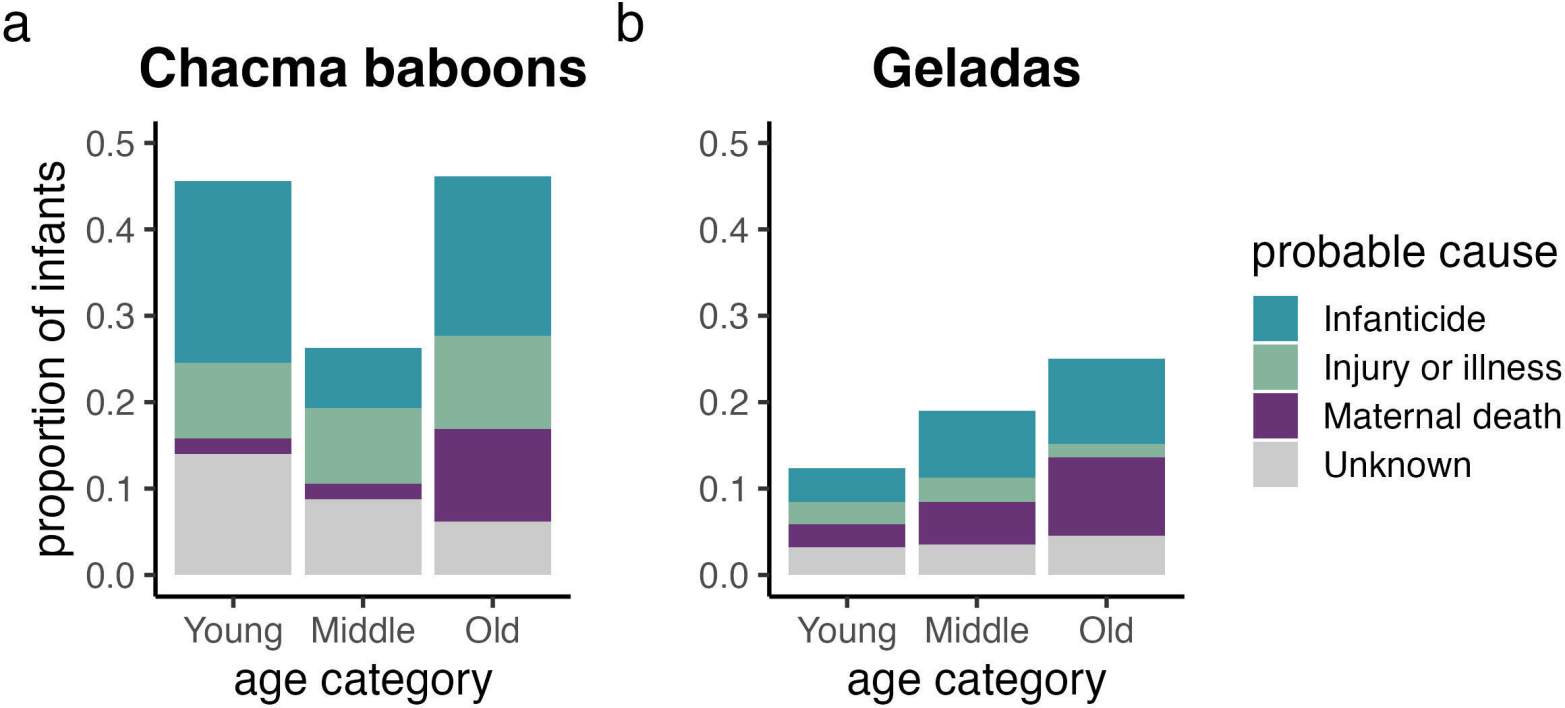
The risk of infant mortality resulting from maternal death—but not infanticide—changed as mothers aged. The proportion of infants (*n* = 179, *n* = 429) that died due to infanticide, injury or illness, maternal death and unknown causes are provided across tertiles of maternal age at birth. Although deaths attributable to infanticide appear to shift across maternal age categories, these trends were not well-supported in either species.

In chacma baboons, the infants of both young and old mothers experienced reduced survival (β_age_ = −0.12 ± 0.13; β_age2_ = 0.35 ± 0.12; figure 2*c*). By contrast, in geladas, infant survival peaked in young mothers and decreased with maternal age (β_age_ = 0.32 ± 0.14; β_age2_ = 0.03 ± 0.09; figure 2*d*). The sources of infant mortality shifted as mothers aged in both chacma baboons and geladas: infant deaths linked with maternal death were more common in old mothers than in mid-aged and young mothers (chacma baboons: β_age_ = 1.14 ± 0.41; geladas: β_age_ = 0.79 ± 0.21: figure 3*a*,*b*). Although infant deaths linked with infanticide were generally more frequent in young and old chacma baboon mothers and in old gelada mothers, neither of these trends had strong empirical support (chacma baboons: β_age_ = −0.06 ± 0.25, β_age2_ = 0.06 ± 0.23; geladas: β_age_ = 0.26 ± 0.23, β_age2_ = −0.04 ± 0.16). Additionally, infant deaths of unknown cause declined with maternal age in chacma baboons but not in geladas (chacma baboons: β_age_ = −0.68 ± 0.33, β_age2_ = 0.24 ± 0.32; geladas: β_age_ = 0.05 ± 0.29, β_age2_ = −0.04 ± 0.23).

## 4. Discussion

Here, we identified salient reproductive ageing trajectories in both chacma baboons and geladas, indicating that high rates of infanticide were not sufficient to obscure these patterns. Instead, reproductive ageing patterns supported the predictions of the ecological hypothesis: chacma baboons showed strong quadratic ageing patterns for both interbirth interval duration and infant survival, and middle-aged mothers showed the highest reproductive performance across the board. In geladas, however, ageing effects were less pronounced and diverged across our two metrics of reproductive success. While both young and old mothers experienced longer interbirth intervals on average, time-varying effects indicate that these two age classes were not equivalently encumbered. Surprisingly, gelada infant survival declined as mothers aged. Further tests revealed that infant deaths linked with maternal death became more common as mothers aged, while infanticide risk was not associated with maternal age. Thus, changes in infanticide risk across maternal age cannot explain these species differences in infant survival trends. Below, we situate these results within our broader understanding of reproductive ageing in primates and other long-lived, social mammals.

### 4.1. Young and old mothers experienced longer interbirth intervals

For both chacma baboons and geladas, young females experienced longer interbirth intervals, which matches previous research across primates and other mammals [29,36,64]. Young or primiparous females generally provide lower-quality maternal care [65,66], wean their offspring at later ages [67,68] and take longer to recoup energetic losses incurred during reproduction [69,70]. In combination, these deficits probably produced the longer interbirth intervals seen in young females of both species. The impact of old age on fertility, however, differed slightly between the two species. Old chacma baboon females experienced long interbirth intervals, presumably due to deteriorating physical condition (e.g. reduced body mass: [71,72]). Few old chacma baboon females failed to close interbirth intervals; however, this could be an artefact of our data inclusion criteria, which reduced the sample from old females (46.9% of interbirth intervals greater than 16 yr removed due to infant death). By contrast, old gelada females appeared to adopt one of two life history strategies: (i) reproduce quickly to produce more offspring within their dwindling lifespans or (ii) slow down reproduction to allocate more resources towards their terminal offspring [22,73]. Further investigations of life history covariation will be necessary to identify such divergent life history strategies and their potential drivers [74].

### 4.2. Old mothers routinely experienced low infant survival

In both species, old mothers experienced lower rates of offspring survival. This was in large part the result of maternal death (i.e. orphaning), which decreases offspring survival in both the short and long term [75,76]. Nevertheless, the maternal deficits associated with advanced age often long precede maternal death [77]. For example, dental senescence can compromise maternal condition and hinder offspring success [78,79]. However, in chacma baboons, senescence-related deficits in maternal condition and behaviour may not directly explain this uptick in infant deaths. Indeed, the vast majority of chacma baboon infant and adult deaths can be respectively attributable to infanticide and predation [38]. Thus, the low rate of infant survival in older chacma baboon mothers might more closely reflect the sum of these extrinsic mortality risks, although reduced physical condition could exacerbate these patterns. By contrast, physical senescence may have a more direct impact on reproductive success in ageing geladas, as predation rates are lower and intrinsic mortality risks may be more salient. Indeed, female geladas from a different study population exhibited signs of reduced chewing efficiency in old age, particularly during the dry season when geladas feed on harder, nutrient-dense foods [80]. Forthcoming measurements of female physical condition in the gelada study population may further capture ageing trends in body mass, dental wear and frailty.

### 4.3. Young mothers showed low infant survival in chacma baboons, but high survival in geladas

Young chacma mothers joined many other mammalian mothers in exhibiting low infant survival, probably due to maternal inexperience [4,81], suboptimal physical condition [3], or other factors that could result in increased infant deaths of unknown causes. By contrast, despite showing long interbirth intervals, young gelada mothers actually showed the highest infant survival rates. Thus, the subcomponents of reproductive output do not always follow parallel ageing relationships. But how might young gelada mothers bypass these infant survival costs? One pathway to doing so might be to delay reproduction until growth is nearly complete, minimizing trade-offs between overlapping growth and reproduction. However, while geladas complete most of their linear growth by the start of reproduction [82], this only marginally exceeds the growth completion observed in primiparous baboons (captive: [83], wild: [84]) which nevertheless experience reduced infant survival [36,85]. Instead, young females may gain this advantage by timing their maturations to the arrival of new males [86], reducing the risk of takeover-related infanticide during their firstborn’s infancy, and thereby driving this age effect. However, infanticide rates were only marginally lower in young gelada mothers, and simulation models suggest that these benefits only emerge in a narrow range of demographic conditions [87]. Thus, this life history strategy cannot fully explain the pattern of increased infant survival.

Alternatively, this pattern could be a by-product of geladas’ graminivorous diet [49]. Perhaps not coincidentally, other primates that exhibit strong, negative relationships between maternal age and infant survival (e.g. sifakas, muriquis: [29,78]) also feed mainly on leafy, non-defensible foods (electronic supplementary material, table S2). As noted above, folivorous primates generally produce smaller infants relative to their body size [44,45], and young mothers may more readily nourish these ‘cheaper’ offspring. Moreover, folivorous primates often show weaker competitive regimes [46,47], in which young females can outrank and outperform their older group-mates (e.g. [88,89]). High infant survival rates in early adulthood could be the result of these two phenomena identified in many folivores. However, these advantages may be limited in their scope. Young folivore mothers rarely achieve faster reproductive rates in addition to higher offspring survival rates [29,90]. Moreover, in other folivorous mammals, reproductive performance variably peaks in early (e.g. red deer: [91]), mid (e.g. sheep: [3,92]) or even later adulthood (e.g. elephants: [15], horses: [17]). Thus, more comparative data—particularly from atelids, colobines and other folivorous mammals (e.g. marsupials, sloths)—are needed to draw links between diet and reproductive ageing patterns, both within primates and across all mammals.

### 4.4. High infanticide risk did not preclude the observation of reproductive senescence

Despite high infanticide risk (chacma baboons: 54%; geladas: 34% of infant deaths), reproductive senescence was apparent in both species. Thus, frequent infanticides (55% of infant deaths: [31]) may not fully explain the lack of empirical ageing effects in wild capuchins [29,77]. Instead, increases in infant mortality during extreme drought periods could obscure these ageing trends [29,30]. However, while infant survival was relatively stable across study years in geladas [93], infant survival rates fluctuated widely in the chacma baboons (22–92% across birth cohorts). Thus, year-to-year environmental changes might not prevent ageing patterns from emerging either. Alternatively, the high levels of allomaternal care observed in capuchins [94,95] could buffer mothers against maternal ageing, mirroring the helper benefits documented in some cooperative breeding birds [20,21]. While allomaternal care has been broadly linked with improved reproductive outcomes [96,97], more research is needed to determine if and how it buffers mothers against the adverse effects of ageing.

More broadly, the data presented here also indicate that reproductive ageing does not follow a uniform pattern, even across closely related species (electronic supplementary material, table S5). While some relationships are common and seemingly conserved (i.e. reduced fertility at young and old ages), others appear to be more evolutionarily or ecologically malleable (i.e. infant survival shortfalls vs. advantages in younger mothers). Indeed, life history traits can be quite plastic and often vary across populations of the same species [98,99], including chacma baboons and geladas [54,100,101]. Thus, the patterns reported here presumably reflect both evolved, species-specific life history traits and local ecological conditions. To better capture the broader drivers of this variation, future studies should focus on both cross-species and cross-population differences, joining datasets representing multiple clades with detailed ecological information and phylogenetic comparative methods.

## Data Availability

Data and relevant code for this research work are stored in GitHub: https://github.com/GeladaResearchProject/Feder_et_al_2024_Reproductive_aging and have been archived within the Zenodo repository [102]. Supplementary material is available online [103].
